# Use of T2 maps for rapid prediction of stress effectiveness before the injection of contrast in myocardial perfusion studies at 3.0T

**DOI:** 10.1186/1532-429X-18-S1-Q53

**Published:** 2016-01-27

**Authors:** Juliano L Fernandes, Luciana A Fioravante, Paulo E Mazo, Andreas Greiser, Ralph Strecker

**Affiliations:** 1 Cardiovascular Imaging, Jose Michel Kalaf Research Institute, Campinas, Brazil; 2 Siemens Healthcare Diagnosticos SA, Sao Paulo, Brazil; 3 Siemens Healthcare GmbH, Erlangen, Germany

## Background

Effective induction of vasodilation during stress perfusion CMR exams may not occur in a clinically significant proportion of patients. While visual analysis of splenic switch-off may offer an accurate assessment of stress adequacy, it can only be analyzed retrospectively. T2 maps may detect stress vasodilatory changes preemptively and allow changes to the protocol prior to contrast injection.

## Methods

Fifty patients undergoing routine stress CMR exams with dipyridamole and a FLASH perfusion sequence were prospectively studied at 3.0 T (Siemens Verio). A single breath-hold myocardial T2 map (TrueFISP sequence, 3 T2 preparation pulses, 9 total heartbeats) of a mid-ventricular short axis slice was obtained using a prototype sequence at rest and immediately after dipyridamole injection, followed by routine perfusion images. T2 values pre and post stress were measured in a ROI of the septum in the inline generated maps. Peak signal intensity (PSI, maximum minus baseline values) of the myocardium in the same region as well in the spleen were calculated. Spleen switch-off was determined visually and semi-quantitatively. Statistical analysis included correlations of the percentage changes of T2 in comparison to differences in spleen PSI and spleen-to-myocardial ratios. ROC curves were used to calculate the accuracy of T2 values to determine the presence of spleen switch-off.

## Results

**Figure 1:**
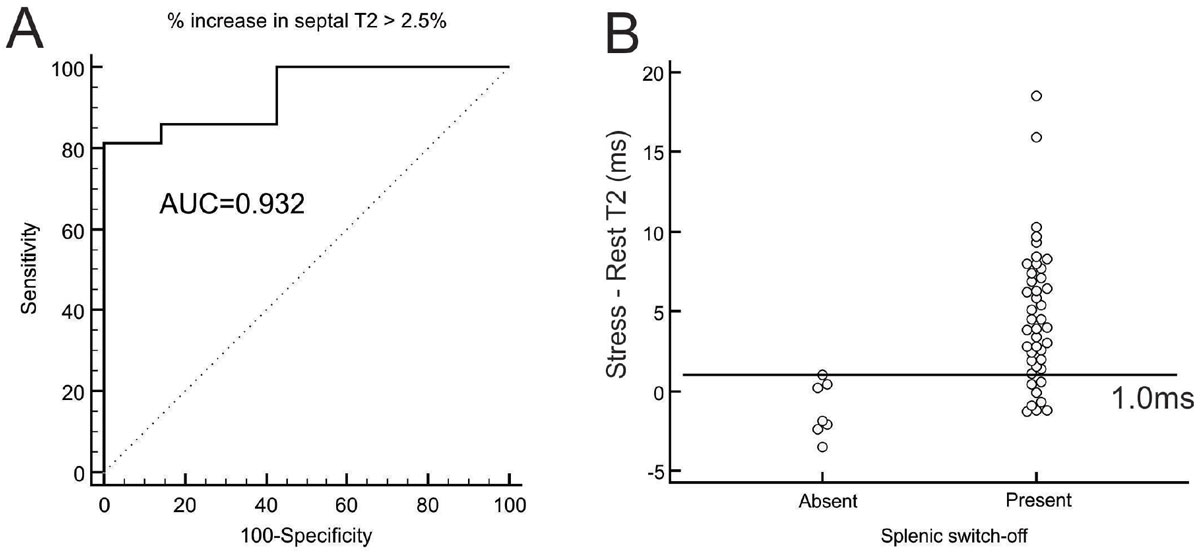
Figure 1

All exams were included in the analysis (62% males, mean age 63.1 ± 11.6 years). The median T2 at rest was 41.2 ms (IQR 39.9-43.5) versus 45.2 ms (IQR 37.7-59.4) during stress (p < 0.001). Patients with absent visual splenic switch-off (n = 7, 14%) had significantly lower stress T2 values compared to patients with positive switch-off (41.1 ms [IQR 40.3-43.3] vs 46.3 [IQR 44.1-48.3], differences of -4.4 ms [IQR-5.5 to 0.8] versus 10.0 ms [IQR 3.8 to 17.6], p < 0.001). The median overall change in spleen PSI was -57.2% (IQR -69.0 to -36.5) with a spleen-to-myocardium ratio at stress of 0.44 (IQR 0.33-0.68). There was a significant correlation of changes in T2 to changes in both parameters (Spearman's rho of -0.51 [95% CI -0.69 to -0.27, p < 0.001] and -0.56 [95%CI -0.73 to -0.33, p < 0.0001] respectively). ROC curve analysis (Figure 1A and 1B) showed that changes > 2.5% or > 1.0 ms in T2 at stress had a sensitivity of 81.4% and specificity of 100% to identify patients with visually positive switch-off (AUC of 0.932 and 0.934 respectively, p < 0.0001 for both criteria).

## Conclusions

Use of T2 maps pre and post stress induction predicts the occurrence of splenic switch-off before the acquisition of perfusion images and may allow for changes in management of the stress protocol improving exam efficacy.

